# Correlating colorectal cancer risk with field carcinogenesis progression using partial wave spectroscopic microscopy

**DOI:** 10.1002/cam4.1357

**Published:** 2018-03-23

**Authors:** Scott Gladstein, Dhwanil Damania, Luay M. Almassalha, Lauren T. Smith, Varun Gupta, Hariharan Subramanian, Douglas K. Rex, Hemant K. Roy, Vadim Backman

**Affiliations:** ^1^ Department of Biomedical Engineering Northwestern University Evanston Illinois 60208 USA; ^2^ Division of Gastroenterology/Hepatology Indiana University School of Medicine Indianapolis Indiana USA; ^3^ Section of Gastroenterology Boston Medical Center/Boston University School of Medicine Boston Massachusetts 02118 USA

**Keywords:** cancer risk, chromatin, colorectal cancer, field carcinogenesis, partial wave spectroscopic microscopy

## Abstract

Prior to the development of a localized cancerous tumor, diffuse molecular, and structural alterations occur throughout an organ due to genetic, environmental, and lifestyle factors. This process is known as field carcinogenesis. In this study, we used partial wave spectroscopic (PWS) microscopy to explore the progression of field carcinogenesis by measuring samples collected from 190 patients with a range of colonic history (no history, low‐risk history, and high‐risk history) and current colon health (healthy, nondiminutive adenomas (NDA; ≥5 mm and <10 mm), and advanced adenoma [AA; ≥10 mm, HGD, or >25% villous features]). The low‐risk history groups include patients with a history of NDA. The high‐risk history groups include patients with either a history of AA or colorectal cancer (CRC). PWS is a nanoscale‐sensitive imaging technique which measures the organization of intracellular structure. Previous studies have shown that PWS is sensitive to changes in the higher‐order (20–200 nm) chromatin topology that occur due to field carcinogenesis within histologically normal cells. The results of this study show that these nanoscale structural alterations are correlated with a patient's colonic history, which suggests that PWS can detect altered field carcinogenic signatures even in patients with negative colonoscopies. Furthermore, we developed a model to calculate the 5‐year risk of developing CRC for each patient group. We found that our data fit this model remarkably well (*R* ^2^ = 0.946). This correlation suggests that PWS could potentially be used to monitor CRC progression less invasively and in patients without adenomas, which opens PWS to many potential cancer care applications.

## Introduction

Field carcinogenesis, first conceptualized by Slaughter et al. 1, is the concept that diffuse molecular and structural alterations exist in healthy tissue prior to the development of a localized tumor. While trying to understand the cause of synchronous or metachronous primary tumors, Slaughter discovered that the benign epithelium outside the tumor boundaries was histologically abnormal. Owing to this observation, he proposed that (1) an oral epidermoid carcinoma originates from a preconditioned field caused by an unknown carcinogenic agent and (2) because the entire field is not removed during surgery, the locally transformed mucosa can give rise to new tumors even after resection (resulting in a high rate of recurrence). Improvements in molecular and genetic analysis have since supported his idea of precancerous fields, showing that there are molecular and genetic alterations within these fields prior to the histologically detectable alterations that Slaughter discovered. The consensus is now that field carcinogenesis is not caused by a single “unknown carcinogenic agent,” but is the result of the gradual transformation of tissue over time due to the complex interaction of many factors including genetic predisposition, environmental exposures, and lifestyle factors, such as smoking and diet 2, all of which have been linked to molecular alterations in the development of cancer 3.

Thus, while there are many different forms and causes of cancer, field carcinogenesis is a ubiquitous early step in the process of carcinogenesis. Notably, cellular exposure to these factors is not always limited to a specific location, but can affect large areas of the body (i.e., smoking increases the risk of lung, bladder, pancreatic, and colorectal cancer). This leads to an important facet of field carcinogenesis: The field is not localized to the tumor site, but can occur across an entire organ, multiple organs, or even the whole body 2. Studies of field carcinogenesis for lung cancer secondary to smoke exposure have shown molecular alterations, such as p53 mutations 4, hypermethylation of multiple genes 5, increased telomerase expression 6, and loss of heterozygosity 7 in histologically healthy epithelial cells of the large airway. Even without chronic exposure to a known carcinogenic agent, studies of field carcinogenesis for colorectal cancer (CRC) have identified biomarkers obtained from the visually normal rectal mucosa showing cellular and molecular changes, such as increased apoptotic resistance 8, increased cell proliferation 9, changed gene expression 10, and modified patterns of protein expression 11. Furthermore, identification of these field biomarkers, such as rectal apoptosis rate measured from normal rectal mucosa, has been shown to be predictive of future adenoma development and could potentially be used to identify high‐risk patients 12.

**Figure 1:**
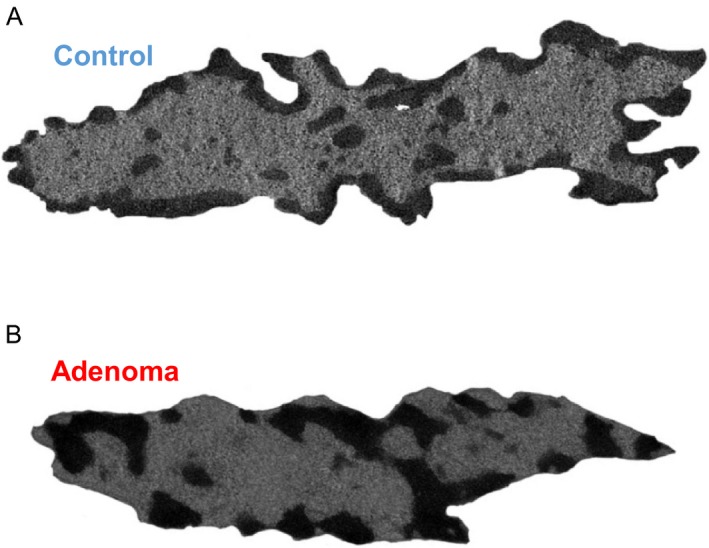
Transmission electron microscopy images of nuclei from endoscopically normal human rectal cells collected from (A) healthy patients and (B) patients with adenomatous polyps. Nanoscale changes in chromatin structure were observed for patients with adenomatous polyps such as increases in heterochromatin content and clump size, as well as a change in the spatial distribution of mass density.

In conjunction with these molecular alterations, a number of early structural alterations in cell nuclei are observed in field carcinogenesis. This is not surprising, as changes in nuclear structure such as size, shape, chromatin texture, and nuclear matrix are gold standard histopathological markers of dysplasia and neoplasia across most cancer types 13. Karyometric studies have found altered nuclear chromatin patterns information in patients with either colorectal adenoma or adenocarcinoma in histologically normal mucosa far from the lesion 14. Using transmission electron microscopy (TEM), our laboratory has observed nanomorphological transformation in chromatin folding, which occur at an earlier stage of field carcinogenesis prior to histologically detectable changes 15 both in (1) preneoplastic human rectal cells from the field of CRC (endoscopically normal rectal mucosa from patients with adenomatous polyps [36 controls and 29 field CRC TEM micrographs]), and (2) rat colon cells at a premalignant time point of the established azoxymethane (AOM)‐injected model of CRC (107 controls and 51 early CRC TEM micrographs). In particular, we observed changes in the higher‐order chromatin structure within these histologically normal cell nuclei showing increased heterochromatin content and clump size, as well as a change in the spatial distribution of mass density (Fig. 1). Analytically, these structural transformations at the micron scale have been shown to be well characterized as a fractal with dimension *D* and utilized as an early marker of tumorigenesis 16. In the nucleus, the fractal scaling as defined by *D* refers to the scaling relationship of chromatin across a given range of length scales for a given physical property (e.g., the geometric relationship between the density of packing nucleosomal fibers into micron scale chromosomal domains).

As these structural alterations converge on length scales of chromatin (<200 nm) that regulate cellular functions (e.g., transcription), we studied the integration of molecular–structural transformations in early carcinogenesis and developed a noninvasive, low‐cost optical method to detect these structural alterations 17. Partial wave spectroscopic (PWS) microscopy provides unprecedented insights into cellular nanoarchitecture at length scales between ~20 and 200 nm by analyzing variations in the backscattered interference spectrum 18, 19, 20. Although we cannot resolve the individual scattering particles within the cell below the diffraction limit, we can measure the resulting changes in light scattering to quantify subdiffractional organization due to the variations in the refractive index 19, 20. As refractive index is a linear function of local macromolecular mass density (DNA, RNA, proteins, etc.), PWS quantifies the nanoscale distribution of mass density in a parameter called disorder strength (*L*_d_), which is sensitive to the topological transformation in chromatin observed by TEM. *L*_d_ is equivalent to $\sigma_{n_{\Delta }}l_{c}$, where $\sigma_{n_{\Delta }}$ is the variance in refractive index fluctuations, and l_c_ is the refractive index correlation length. Physically, nuclei with increased *L*_d_ have chromatin configurations that are more globally accessible paired with highly dense, local clumps of poorly accessible chromatin. Thus, the structural transformation captured by PWS at the nanoscale mirrors the fractal transformation observed at later stages in carcinogenesis. As higher‐order chromatin folding has been shown to be well characterized as a fractal media at these length scales (>10 nm), this suggests PWS microscopy is quantifying the topological transformation of this folding in early carcinogenesis 21, 22, 23. Further, we have demonstrated a link between the physical transformation of higher‐order chromatin folding as measured by *L*_d_ and known molecular regulators of chromatin structure often transformed in carcinogenesis. Functionally, consequences of this distortion in chromatin folding have been shown in vitro to result in global changes in gene expression, with increased topological heterogeneity corresponding to transcriptional heterogeneity and divergence in expression 24. In this context, the convergence of multiple molecular regulators on physical folding and global transformation in gene expression suggests that increases in the heterogeneity of chromatin structure could lead to an increase in genomic sampling and facilitate tumor formation 24, 25.

We have established the sensitivity of PWS to nanoscale alterations in colon carcinogenesis through genetic perturbations in colon cancer cell lines, animal models, and in human samples 18, 26. In all cases, PWS measurements were well correlated with the aggressiveness or neoplastic potential of these morphologically normal‐appearing cells, even correlating to the size of the adenoma in humans. Further, evidence of this transformation extends into other cancers, including lung, pancreas, ovarian, esophagus, and prostate 19, 27, 28, 29, 30, 31. Yet to be established, however, is whether PWS is sensitive to CRC risk by more than just the current presence of an adenoma. To do so, we developed a model of CRC risk from a meta‐analysis of published data for each of our patient groups that take into account the history and current colon health of each patient. Pairing this meta‐analysis with PWS measurements, we explore the progression of nanoscopic alterations in chromatin during field carcinogenesis by measuring samples collected from patients with a range of colonic history (no history, low‐risk history, and high‐risk history) and current colon health (healthy, nondiminutive adenoma [NDA], advanced adenoma [AA]). We show that PWS microscopy is sensitive to the severity of a patient's colonic history and that nanoscale alterations in chromatin strongly correlate with CRC risk. These results support the theory that PWS is directly measuring field carcinogenesis, which mirrors the risk of developing cancer. As a low‐cost (~$150), noninvasive technique that can directly measure field carcinogenesis, PWS has many important potential applications such as colonoscopy surveillance and chemoprevention.

## Materials and Methods

### Partial wave spectroscopy (PWS) instrumentation and measurements

A complete description of the PWS instrument and theory used for this study can be found in these references 19, 20. In brief, a spatially incoherent white light (Xenon lamp, 66902 150 W; Oriel Instruments, Stratford, Connecticut, USA) illuminates a specimen, and the backscattered image is projected through a liquid crystal tunable filter (LCTF; CRi, Woburn, Massachusetts, USA; spectral resolution = 7 nm) onto a CCD camera (Princeton Instruments, Trenton, NJ, USA). Our signal originates from the interference between the scattering from various structures and macromolecular complexes within a cell and the strong reflection from the interface at the top surface of the cell, producing spectral fluctuations (interference spectra) in the collected backscattered light. The LCTF and CCD capture a series of microscope images, one image for each wavelength between 500 and 700 nm. The resulting data are stored in an image cube (*x*,* y*, λ), where an interference spectra are stored for each pixel position (*x*,* y*) within the field of view. *L*_d_ is calculated from the fluctuations in the interference spectra as detailed in Cherkezyan et al. 20.

### Clinical sample preparation

All studies were performed, and samples were collected with the approval of the Institutional Review Board at NorthShore University Health System, University of Chicago, and Indiana University Medical Center. Patients undergoing screening or surveillance colonoscopy were included in the study. The exclusion criteria included incomplete colonoscopy (failure to visualize cecum), poor colonic preparation, coagulopathy, prior history of pelvic radiation, or systemic chemotherapy. The samples were collected in a consistent manner as follows: colonoscopy to cecum was performed with standard techniques using Olympus 160 or 180 series or Fujinon colonoscopes. Upon insertion of the colonoscope into the rectum, a sterile cytology brush was passed through the endoscope and gently applied to the visually normal rectum. A single cytology brush was used for each patient. This brush was smeared onto two glass slides, which were then fixed in 95% ethanol, measured using PWS, and analyzed. This entire process is performed by an investigator blinded to the patient information (Analysis Reproducibility in Appendix S1). Slides were examined under a bright field microscope to determine adequate quality (i.e., at least a few colonocyte tissue beds, free from debris, and mucus). Samples of insufficient quality were removed from the study. All the measurements reported here were taken from columnar epithelium (i.e., colonocytes) as identified by standardized hematoxylin and cytostain staining and randomly selected from the regions not hindered by mucus or cell debris.

### Statistical methods

*P*‐values were calculated using two‐tailed Student's *t*‐test with unequal variance (Student's *T*‐Test and Normality in Appendix S1). In this study, effect sizes were calculated using Cohen's d, which is defined as the difference between two means divided by the pooled standard deviation. Percent differences were calculated by dividing the difference between two means by the average of those means. On average, 40 cells were measured and analyzed per patient. By measuring ~40 cells per patient, the average error for each patient *L*_d_ was <2% of the interpatient variability (average intrapatient standard error divided by the interpatient standard deviation). All the parameters were calculated using Microsoft Excel (Microsoft Corporation, Redmond, Washington, USA). STATA (StataCorp LP, College Station, Texas, USA) software was used to calculate ANCOVA (analysis‐of‐covariance) to determine contributions of demographic factors toward *L*_d_.

### CRC risk model

We developed a model to calculate the 5‐year cumulative risk of developing CRC for each of our patient groups using a meta‐analysis of values obtained from the literature. In brief, for patients with no history or history of adenoma, the risk of developing future advanced adenoma is calculated based on data obtained from a surveillance colonoscopy study 32. Then, the risk of AA to CRC progression is determined from additional screening colonoscopy studies 33. The risk for patients with a history of CRC is based off the risk of developing metachronous CRC 34. The full model is explained below.

$$\begin{array}{cc} & \text{Group CRC risk}=\\ & \left(\frac{1}{N_{a}+N_{c}}\right)\left({\text{AA}}_{r}\times \sum_{i=1}^{N_{a}}{\text{AA2CRC}}_{i}+N_{c}\;\times \;{\text{CRC}}_{m}\right) \end{array}$$

where *N*_a_ is the number of patients in the group with either no history or history of adenoma, *N*_c_ is the number of patients in the group with a history of CRC, AA_r_ is the risk of developing future advanced adenoma for each patient group, AA2CRC is the cumulative risk of AA to CRC progression for each patient (based on age and gender), and CRC_m_ is the cumulative risk of developing metachronous CRC.

In detail, AA_r_ (risk of developing future advanced adenoma for each patient group) was determined based off a study by Pinsky et al. 32. Patients within this study had a baseline colonoscopy and then two follow‐up surveillance colonoscopies over a 10‐year period to observe how adenoma findings at the baseline and first surveillance colonoscopy influence AA rates at the second surveillance colonoscopy. Patients within this surveillance study are broken into groups almost identical to our study, if we consider the baseline colonoscopy to be the patients’ colonic history and the first surveillance colonoscopy to be the patients’ current colonic health. The percentage of patients who developed AA at the second surveillance colonoscopy produces a risk of developing future AA within ~3.4 years (average time between first and second surveillance colonoscopy) for each of the patient groups in our study. The only discrepancy in this dataset is that our low‐risk/nondiminutive groups only include adenomas ≥5 mm and <10 mm, while the surveillance colonoscopy study 32 includes all adenomas <10 mm in their lower‐risk group.

Next, to convert this to CRC risk, the cumulative risk of AA to CRC progression (AA2CRC) was calculated for each patient group based on data from Brenner et al. 33. Brenner shows that risk depends on both age and sex. Therefore, the annual AA to CRC progression risk was determined individually for each patient within our study based on their age and sex. This annual risk was converted to a cumulative risk using the formula below.

$$\text{Cumulative risk}=1-e^{-\mathrm{annual}\;\mathrm{risk}\times \mathrm{time}}.$$

As the risk of developing AA took place over 3.4 years, the time period for cumulative risk of progressing from AA to CRC was set to 1.6 years to determine the total risk of developing CRC over 5 years for each of our patient groups. These individual progression risks were then averaged for each patient grouping to account for the different makeup of each group.

Patient groups with high‐risk history contain patients with a history of both advanced adenomas and CRC. Patients with CRC were not included in the surveillance study 32 used to calculate the AA risk; therefore, we calculated risk separately for patients with a history of CRC. For CRC history patients, we used a 0.35% annual risk of developing metachronous CRC 34. This was converted to 5‐year cumulative risk using the formula above. Finally, the risk of patients with a history of CRC and history of AA (using the original method) was combined using a weighted average based on the number of each type of patient within the high‐risk history groups.

It should be noted that while we did not separate our patients based on the number of detected adenomas, the potentially increased risk from multiple adenomas is taken into account in this model through the data from Pinsky et al. 32. (Multiple Adenomas in Appendix S1).

## Results

**Figure 2:**
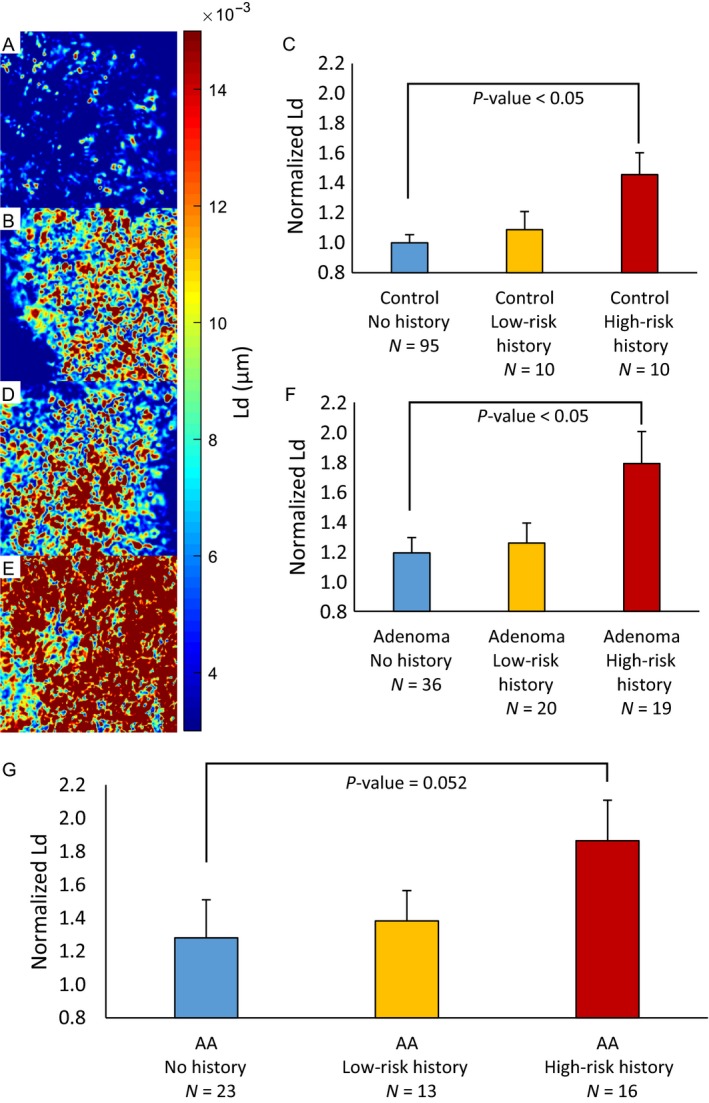
Partial wave spectroscopic (PWS) *L*_d_ map from tissue bed of rectal colonocytes from patients (A) Control No History, (B) Control High‐Risk History, (D) Adenoma No History, and (E) Adenoma High‐Risk History. (C) Within the control group, *L*_d_ increases as the patient's level of history risk increases (Control No History vs. Control High‐Risk History [*N* = 95 vs. 10, percent difference = 37.2%, *P *=* *0.013, effect size = −0.866]). (F) Within the adenoma group, *L*_d_ increases as the patient's level of history risk increases (Adenoma No History vs. Adenoma High‐Risk History [*N* = 36 vs. 19, percent difference = 40.2%, *P *=* *0.019, effect size = −0.807]). (G) There is a large difference between advanced adenoma patients (the most clinically significant, highest risk category) with high‐risk history and no history (*N* = 23 vs. 16, percent difference = 37.1%, *P *=* *0.052, effect size = −0.698). These results show that PWS is sensitive to the history of a patient's colon, which suggests that although these patients have clinically similar colons as determined by colonoscopy, patients with high‐risk history actually have elevated and mutationally active field carcinogenesis that could lead to future colorectal cancer.

To evaluate if PWS is sensitive to field carcinogenesis not connected to active levels of dysplasia, samples were collected from patients with a history of adenomas or CRC. As described above, these patients are likely to have a recurrence of adenomas, which can lead to future CRC. Tissue samples from 190 patients were collected from histologically normal regions of the rectum by cytological brushing and measured using PWS microscopy. These patients have been broken up into six groups based on the current state of the colon, control versus adenoma (≥5 mm), and the patient's history (no history, low‐risk history, and high‐risk history). The low‐risk history groups include patients with a history of nondiminutive adenomas (NDA; ≥5 mm and <10 mm). The high‐risk history groups include patients with either a history of advanced adenoma (AA; ≥10 mm, HGD, or >25% villous features), or a history of CRC. We observed that PWS is sensitive to the history of the patient's colon as well as the current state. Within the control group (patients without a present adenoma), we see a trend in which *L*_d_ increases as the patient's history risk level increases: control no history < control low‐risk history < control high‐risk history (Fig. 2A,B,C). There is a significant difference between control patients without history of adenoma and those with high‐risk history (*N* = 95 vs. 10, percent difference = 37.2%, *P* = 0.013, effect size = −0.866). This trend is also true for patients with current adenomas: adenoma no history < adenoma low‐risk history < adenoma high‐risk history (Fig. 2D,E,F). There is a significant difference between adenoma patients with and without high‐risk history (*N* = 36 vs. 19, percent difference = 40.2%, *P* = 0.019, effect size = −0.807). Nondiminutive adenomas can be removed from this dataset to compare *L*_d_ values for patients with current advanced adenomas—the most clinically significant—highest risk category (CRC progression risk of ~2–6% per year 33; Fig. 2G). There is a large difference between advanced adenoma patients with high‐risk history and no history (*N* = 23 vs. 16, percent difference = 37.1%, *P *=* *0.052, effect size = −0.698). These findings are significant as it suggests that although these patients have similar findings via their current colonoscopy, they actually have a more distorted field that can change their risk for future neoplastic transformation as represented by their increased *L*_d_ (Table 1). Consistent with our previous studies, within each history risk category patients with current adenomas have higher *L*_d_ values than controls, for example: control no history < adenoma no history (*N* = 95 vs. 36, percent difference = 17.4%, *P *=* *0.108, effect size = −0.342, Table 1). The full table and box plots of group comparisons are available in Table S1 and Figure S1, respectively.

**Table 1:** Important group comparisons. This table reports the *p*‐value of the Student's *t*‐test, effect size, and percent difference for important patient group comparisons

Patient groups		*T*‐test	Effect size	% Difference
Control No history	Control, high history	0.0131	−0.866	37.2
	Adenoma, no history	0.108	−0.342	17.4
Control Low history	Adenoma, low history	0.3570	−0.314	14.4
Control High history	Adenoma, high history	0.2120	−0.410	20.5
Adenoma No history	Adenoma, high history	0.0185	−0.807	40.2
Advanced adenoma No history	Advanced adenoma, high history	0.0522	−0.698	37.1

**Figure 3:**
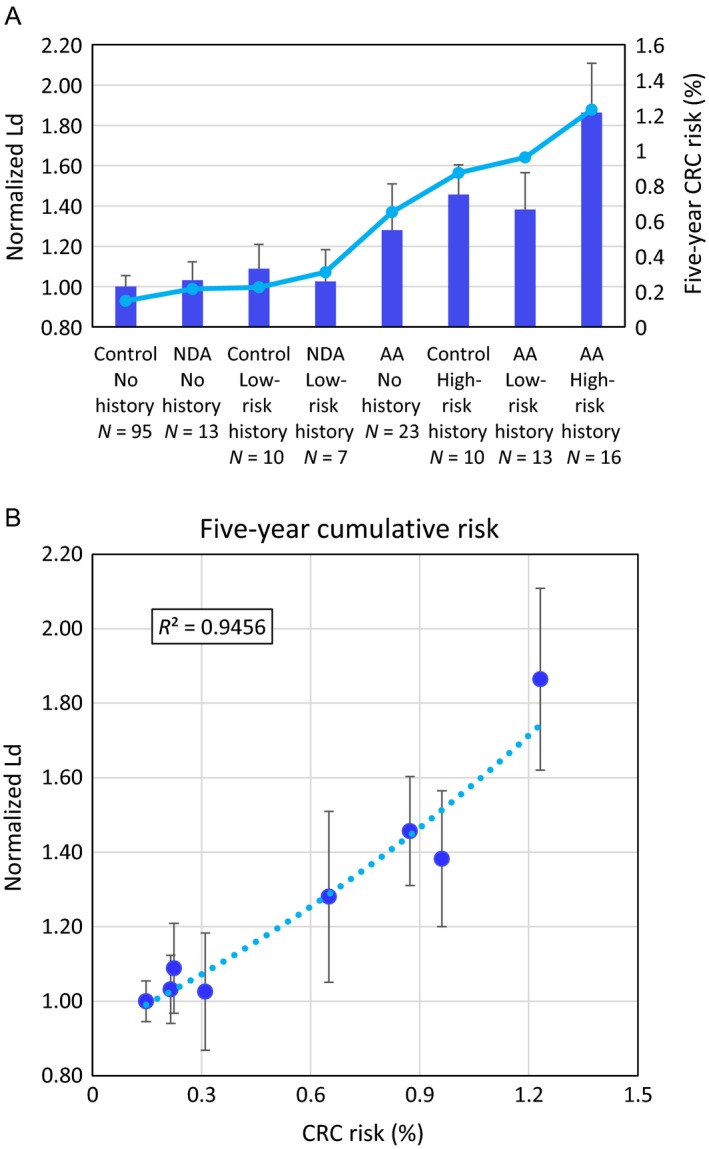
*L*_d_ correlated with calculated risk of developing colorectal cancer (CRC). (A) Bar plot of *L*_d_ values compared to 5‐year cumulative CRC risk with current adenoma groups split into nondiminutive adenomas and advanced adenomas. (B) Scatter plot showing correlation between *L*_d_ and 5‐year cumulative risk of developing CRC (*R* ^2^ = 0.946). This strong correlation between partial wave spectroscopic (PWS) measurements and CRC risk strengthens the argument that these alterations in nanoscale chromatin organization are indeed a feature of field carcinogenesis. Additionally, it suggests that PWS could potentially be used to monitor a patient's CRC risk at an early state without adenomas present.

To see how well our PWS measurements of field carcinogenesis match real CRC risk, we have calculated the 5‐year cumulative risk of developing CRC for each of our groups using a model we developed from a meta‐analysis of the current literature (see CRC risk model section). The number of patient groups were increased to eight in order to get a better sense of how well this model fit our data by splitting the current adenoma groups into current nondiminutive adenoma and advanced adenoma (nondiminutive adenoma with high‐risk history was not included due to only having three patients in that group). An exponential fit was used to describe the relationship between *L*_d_ and CRC risk, and *R* ^2^ was calculated to determine how well the data fit the model (5‐year cumulative risk *R* ^2^ = 0.946, *F* = 104, *P *=* *5.15 × 10^−5^; Fig. 3). This correlation between PWS measurements and CRC risk strengthens the argument that these alterations in nanoscale chromatin organization are indeed a feature of field carcinogenesis and suggests that PWS can potentially be used to monitor CRC disease progression earlier and less invasively than current techniques.

Of note, from this breakdown, we can see that *L*_d_ is much more affected by advanced adenoma—both history and current—than nondiminutive adenomas. This is consistent with our calculated CRC risk as well as other studies which have shown that patients with a nondiminutive adenoma are much less likely to develop an advanced adenoma (8.7% vs. 16.6%) or CRC (0.5% vs. 0.9%) after polypectomy than those with advanced adenoma 35. One potentially confusing comparison is the large difference seen between control patients with high‐risk history and adenoma patients with no history (*N* = 10 vs. 36, percent difference = 20.1%, *P *=* *0.154, effect size = 0.451). This is largely explained by the inclusion of CRC patients in the high‐risk history and nondiminutive adenoma patients included in the current adenoma group. If all of those patients are removed, the difference between these groups becomes quite small (*N* = 6 vs. 23, percent difference = 4.9%, *P *=* *0.791, effect size = 0.09).

To account for confounding variables, we evaluated the impact of demographic risk factors such as age, gender, smoking, and drinking history on the trends reported in this study. Age has been found to be well correlated with colonic neoplasia, and there have been a number of age‐related changes in colonic mucosa (e.g., methylation 36). We performed ANCOVA analysis on our data and noted no significant confounding with age (*P *=* *0.597). Furthermore, smoking and drinking history also did not have any significant confounding effect (*P *=* *0.968 for smoking and *P *=* *0.657 for drinking). Our ANCOVA analysis did not indicate any significant confounding with gender (*P *=* *0.334). Overall, the nonsignificant ANCOVA *P*‐values suggest that the results discussed above are not confounded by age, gender, smoking, or drinking patterns (Table 2). Additional analysis on the impact of these demographic risk factors on *L*_d_ can be found in the Appendix S1 (Confounding Factors).

**Table 2:** Confounding factors. Analysis‐of‐covariance (ANCOVA) analysis has been performed to see whether the results reported in this study are confounded by demographic factors

Demographic factors	Control no history	Control low‐risk history	Control high‐risk history	Adenoma no history	Adenoma low‐risk history	Adenoma high‐risk history	Effect on *L*_d_ ANCOVA *P*
Age (mean ± SD)	55 ± 11	66 ± 6	63 ± 16	61 ± 9	67 ± 12	68 ± 11	0.597
Gender (% male)	47	70	40	47	65	42	0.334
Current smokers (%)	12	22	20	8	20	0	0.968
Former smokers (%)	23	22	30	31	45	32	
Alcohol Users (%)	68	100	20	44	60	37	0.657

Overall, the nonsignificant ANCOVA *P*‐values suggest that the results of this study above are not confounded by age, gender, smoking, or drinking patterns.

## Discussion

Field carcinogenesis is one of the earliest events in the process of carcinogenesis and a common denominator of multiple molecular pathways, convolving personal genetics, carcinogen and microbial exposures, and lifestyle factors 2. Although the cells within the field are histologically normal in early carcinogenesis, they contain cellular, molecular, and nanostructural alterations 8, 9, 10, 11, 15. Previous results have indicated that nanoscale chromatin alterations are an early and ubiquitous event in the development of field carcinogenesis within many different cancer types (colorectal, lung, pancreas, ovarian, esophagus, and prostate 19, 27, 28, 29, 30, 31). In this work, we show that nanoscopic changes in chromatin folding are highly correlated with CRC risk and support the theory that PWS directly measures field carcinogenesis and cancer risk.

**Figure 4:**
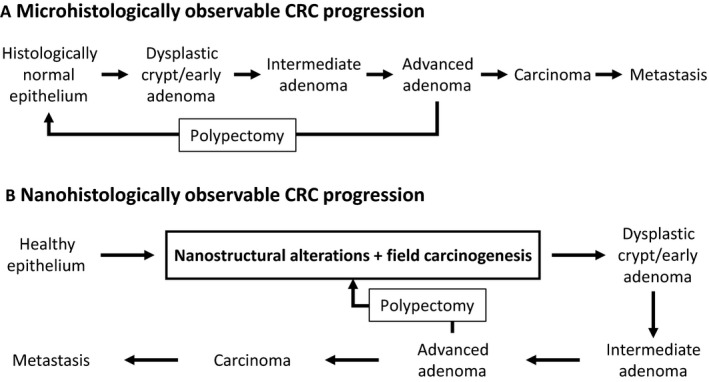
(A) A diagram of the histologically observable disease progression of colorectal cancer. In this progression, polypectomy brings the patient back to the initial healthy state. (B) Incorporating nanostructural alterations and field carcinogenesis into the colorectal cancer (CRC) progression provides an additional framework to explore cancer progression and detection. In this framework, polypectomy reduces risk of CRC progression and returns the patients to a microscopically healthy state, but there are persistent nanostructural and molecular alterations that maintain an elevated CRC risk compared to a completely healthy patient.

Classically, the assumption has been that removal of AAs found during colonoscopy brings the patient back to the first stage in the progression of CRC (Fig. 4A). While the colon appears histologically healthy after adenoma resection, our data indicate the persistence of nanoscopic changes in chromatin that are associated with recurrence risk (Fig. 4B). While the molecular mechanisms are not explored within this work, our results suggest a continuum of nanoscale states preceding the reformation of histologically observable progression. As reported above, control patients with AA history and current AA patients with no history show no difference (*N* = 6 vs. 23, percent difference = 4.9%, *P *=* *0.791, effect size = 0.09). This can be understood in the context of the development of cancer as a continuous probabilistic process. Each step in the progression from healthy tissue to metastatic cancer is a localized stochastic event that occurs within the diffuse field of the organ. The correlation between CRC risk and field carcinogenesis as measured with PWS indicates that the severity of the field determines the probability of these events reoccurring. While polypectomy will decrease the immediate risk of developing a neoplastic lesion, it does not seem to change the probability of the early events reoccurring.

While this data suggests that removal of an adenoma does not change the nanoscopic transformation of the colon, it is not known if interventions can revert the colon toward a nanoscopically healthy state over time. This is an interesting question that PWS might be able to answer in the future. It seems logical that such interventions may exist because there are factors that reduce CRC risks such as diet, exercise, and therapeutics (i.e., aspirin) 2, 37. There has been some research into this question by Avrum Spira et al. 38, who looked at changes in gene expression in airway epithelial cells collected from healthy patients, smokers, and former smokers. By comparing the expression of 97 genes that are altered in smokers compared to never smokers, they observed that patients who have discontinued smoking for >2 years are more closely clustered with never smokers than current smokers. This data suggests that some changes in field are reversible, but there were several tumor suppressor genes and potential oncogenes which did not revert to normal 38. Additionally, we observed normalization of different nanostructural field carcinogenic markers in the colon in vivo after aspirin treatment using an alternative optical technique called LEBS 39.

If the progression of the carcinogenic field can be reverted to a healthier state, PWS should be able to detect those changes, which could lead to developing chemopreventive therapeutics. Chemopreventive drugs have the potential to save many lives, but they are difficult to develop and test as there are no reliable markers of efficacy. PWS measurements of field carcinogenesis have the potential to provide this missing marker. To test this, a larger retrospective analysis of nanoscopic changes within the colon would include patient medication data in comparison with the expected risk outcome based on their history. Compounds which decrease the nanoscale transformation of chromatin and correlate with decreased recurrence risk would then be screened on induced animal models (e.g., AOM rats). Depending on the outcome of such a study, PWS could be used to personalize treatment by sensing if a specific drug and dosage are effective for that patient similar to the LEBS study mentioned above 39. As an example, nonsteroidal anti‐inflammatory drugs, such as aspirin, have shown chemopreventive effects in numerous studies 40, 41. Specifically, aspirin has been shown to reduce adenoma recurrence 40 and incidence of CRC 41. Unfortunately, aspirin will result in a chemopreventive benefit for only 30–50% of patients and has serious side effects including gastrointestinal bleeding and hemorrhagic stroke 39, 41. Therefore, chemopreventive use of aspirin is not recommended in patients unless they are known to have a high risk of developing cancer 37. If we could differentially predict aspirin efficacy, then we could reduce these unnecessary side effects to nonresponders.

Another potential application for PWS based on the findings within this work is to aid in colonoscopy surveillance. CRC is the second leading cause of cancer‐related deaths in the United States with 134,490 new cases and 49,190 deaths estimated for 2016 42. The number of incidences and deaths remain quite high despite the effectiveness of colonoscopy via removal of adenomatous polyps 43. One barrier to reducing CRC incidence is the reluctance of patients to undergo colonoscopy because of cost, complications, discomfort, and insufficient allocation of resources. Colonoscopy surveillance is recommended because a previous history of adenoma is an important risk factor for future neoplasia, but the current guidelines are empirically derived and suboptimal. Additionally, some studies suggest that adenoma‐based risk stratification has limited predictability for advanced adenomas 44. As a result, many clinicians ignore these guidelines, which cause insufficient allocation of resources 45. Approximately 90% of postpolypectomy colonoscopies are negative for screening relevant neoplasia 35, and it is estimated that 317 colonoscopies are required to detect a single case of CRC 46. Further complicating the issue is that colonoscopy provides insights into risk over a finite time period and not necessarily the more important lifetime risk. This leads to very serious issues such as interval cancers. Studies have shown that ~8% of CRC patients had a negative colonoscopy 6–36 months before diagnosis 47. Clinically, determining an optimal surveillance interval is a challenge between overutilization of colonoscopy with the corresponding cost and potential complications versus too long an interval with the risk of interval cancers. PWS is a potential solution to this problem. First, PWS measurements could be taken prior to colonoscopy to identify patients with the highest CRC risk and therefore most likely to benefit from a colonoscopy. Alternatively, PWS measurements could be taken at the same time as colonoscopy to help determine optimal surveillance intervals. This may reduce interval cancers by identifying patients who are at high risk for CRC, even when no adenoma is found during colonoscopy. This is because colonoscopy can only determine if a patient currently has an adenoma while PWS, by measuring the chromatin transformation during field carcinogenesis, is sensitive to both the current and past state of the colon and is likely sensitive to other cancer risk factors such as family history, exogenous carcinogen exposure (e.g., smoking and occupational or chemical exposures), and dietary history. As a low‐cost, noninvasive technique that can directly measure field carcinogenesis, PWS thus has the potential to assist and optimize the use of colonoscopy.

### Limitations

We would like to acknowledge the limitations of this study. First, while we have been able to show that nanoscale chromatin heterogeneity is correlated with the disease progression of field carcinogenesis, the suggested applications of this technique (surveillance and drug efficacy) are extrapolations because this is a retrospective study. A prospective study that follows *L*_d_ values over time for the same patients is necessary to validate these applications. Additionally, while the CRC risk model is based on credible clinical data, it should be acknowledged that there have been some discrepant clinical studies. For example, Laiyemo et al. 44, showed that while the risk of AA recurrence is greater for patients with a history of AA compared to those with low‐risk adenomas, the differences between these two groups are smaller than reported in other studies. Finally, it should be noted that the insignificant *P*‐values obtained from ANCOVA analysis used to check for confounding factors could be due to the small number of patients in some groups. There is additional testing for confounding factors in the Appendix S1 (Confounding Factors).

## Conclusions

This study has shown that alterations in nanoscale chromatin organization are a feature of field carcinogenesis and that PWS has the potential to monitor CRC progression risk. It should be noted that these results are not necessarily specific to CRC; it is possible that they are valid for many different types of cancer. As mentioned above, PWS has already been shown to detect field carcinogenic signatures in cancers such as lung, pancreas, ovarian, esophagus, and prostate 19, 27, 28, 29, 30, 31. As a noninvasive tool that can monitor this progression, the functionality of PWS has been greatly expanded toward many new uses. Two examples, colonoscopy surveillance and chemopreventive drug efficacy, are given in this study.

## Conflict of Interest

Drs. Roy and Backman are co‐founders/shareholders of NanoCytomics LLC.

## Supporting information


**Figure S1**. Box plots showing normalized *L*_d_ values increasing based on the patient's risk history for (a) current controls patients, (b) current adenoma patients, and (c) current advanced adenoma patients.
**Figure S2**. Additional testing of the effects of confounding factors on *L*_d_.
**Figure S3**. This normal probability plot for control patients, no history (*n* = 95) shows that the *L*_d_ distribution is slightly skewed to the left.
**Figure S4**. Scatter plot comparing patient *L*_d_ values analyzed (cell selection and drawing ROIs) by two different investigators (*R* ^2^ = 0.97).
**Table S1**. Full set of group comparisons.
**Appendix S1**. Supplemental information containing additional analysis of confounding factors, explanation of patients with multiple adenomas included in this study, analysis on the normality of our data and the use of Student’s t‐test, and data on the reproducibility of PWS analysis.
