# Supplementary material for: Correlating colorectal cancer risk with field carcinogenesis progression using partial wave spectroscopic microscopy
Source: Cancer Med. 2018 Mar 23;7(5):2109–20. doi: 10.1002/cam4.1357 (PMC5943438; doi:10.1002/cam4.1357)
Supplement: Supplementary file 1 — Figure S1. Box plots showing normalized L d values increasing based on the patient's risk history for (a) current controls patients, (b) current adenoma patients, and (c) current advanced adenoma patients. Figure S2. Additional testing of the effects of confounding factors on L d. Figure S3. This normal probability plot for control patients, no history (n = 95) shows that the L d distribution is slightly skewed to the left. Figure S4. Scatter plot comparing patient L d values analyzed (cell selection and drawing ROIs) by two different investigators (R 2 = 0.97). Table S1. Full set of group comparisons. Appendix S1. Supplemental information containing additional analysis of confounding factors, explanation of patients with multiple adenomas included in this study, analysis on the normality of our data and the use of Student’s t‐test, and data on the reproducibility of PWS analysis. [file CAM4-7-2109-s001.pdf]

Supplementary Information

Confounding Factors

It is possible that the insignificant p-values obtained from ANCOVA analysis used to check for confounding factors in the main text could be due to the small number of patients in some of the groups. Additional testing was performed by combining all patient groups to determine if there is a general relationship between these potential confounding factors and L_d. There is no visible trend when comparing the relationship between L_d and age (R^2=0.03) (**SI Figure 2A**). Additionally, there was no significant difference in L_d between male and female patients (n = 94 vs 96, p-value = 0.402), never smokers compared to both current smokers (n = 113 vs 22, p-value = 0.914) and former smokers (n = 113 vs 52, p-value = 0.431), and patients who drink alcohol compared to those who do not (n = 109 vs 78, p-value = 0.131) (**SI Figure 2BCD**).

Multiple Adenomas

In this study, patient groupings were determined by the size and histology of the adenomas detected during colonoscopy. While some studies separate patients based on the number of adenomas, this was not done in this study in order to be consistent with the data used to develop the CRC risk model. The data from Pinsky et al. was used to determine the risk of developing future AA for each of our patient groups in the CRC risk model. Pinksky et al. included patients with multiple adenomas in their study, but did not use that information to classify their patients. By following the same strategy, our study already takes into account the potentially altered risk from patients with multiple adenomas. Furthermore, we confirmed that our patients have a typical distribution of the number of adenomas at their most current colonoscopy compared to multiple literature sources (49.3% of patients have 1 adenoma, 31.5% of patients have 2 adenomas, 9.6% of patients have 3 adenomas, 5.5% of patients have 4 adenomas, and 4.1% of patients have 5 or more adenomas)[1-3].

Student's T-Test and Normality

The significance of group differences reported in this paper were calculated using a two-tailed Student's t-test with unequal variance. The Student's t-test assumes normally distributed data. To test normality in our dataset, we have created a normal probability plot and calculated the Anderson-Darling test. These tests for normality are far more accurate with larger samples sizes, so they were performed on our control no history group, which has the largest samples size (n=95). The normal probability plot shows that the data is slightly skewed to the left (**SI Figure 3**). The Anderson-Darling test for a normal distribution produces a p-values of 0.051. This means that the test cannot reject the null hypothesis at the 5% significance level, but it is close. Taken together, this data suggests that the distribution may not be perfectly normal, but it is close to normal. This should not be such an issue for the Student's t-test, which is relatively robust to non-normal data [4]. To be thorough we have performed the Mann-Whitney U-test, a nonparametric test that determines if two independent samples were selected from populations

with the same distribution; it does not require the assumption of a normal distribution. Data comparing the significance of the Student's t-test and the Mann-Whitney U-test are included in **SI Table 1**. In summary, the significant important comparisons highlighted in the manuscript figures are still significant with both the Student's t-test and the Mann-Whitney U-test. There are some less important subgroup comparisons that become less significant, while others become more significant when using the Mann-Whitney U-test.

Analysis Reproducibility

To test reproducibility in analysis, two different investigators blindly chose cells and drew regions of interest from the same patient data. Over 39 patients, L_d values analyzed by these different investigators showed a high degree of correlation ($R^2 = 0.97$) (**SI Figure 4**).

Supplemental References

1. Martinez ME, Baron JA, Lieberman DA, Schatzkin A, Lanza E, Winawer SJ, Zauber AG, Jiang R, Ahnen DJ, Bond JH, Church TR, Robertson DJ, et al. A pooled analysis of advanced colorectal neoplasia diagnoses after colonoscopic polypectomy. *Gastroenterology* 2009;**136**: 832-41.
2. Jorgensen OD, Kronborg O, Fenger C, Rasmussen M. Influence of long-term colonoscopic surveillance on incidence of colorectal cancer and death from the disease in patients with precursors (adenomas). *Acta oncologica (Stockholm, Sweden)* 2007;**46**: 355-60.
3. Schoen RE, Pinsky PF, Weissfeld JL, et al. Results of repeat sigmoidoscopy 3 years after a negative examination. *JAMA* 2003;**290**: 41-8.
4. Lumley T, Diehr P, Emerson S, Chen L. The importance of the normality assumption in large public health data sets. *Annual review of public health* 2002;**23**: 151-69.

Supplemental Tables

SI Table 1.

Patient Groups		Student's T-Test	Mann-Whitney U Test	Effect Size (Cohens D)	% Difference
Control No History	Control, Low History	0.5156	0.5740	-0.170	8.5
	Control, High History	0.0131	0.0119	-0.866	37.2
	Adenoma, No History	0.108	0.1383	-0.342	17.4
	Adenoma, Low History	0.0867	0.1371	-0.473	22.8
	Adenoma, High History	0.0019	0.0009	-1.281	56.6
Control Low History	Control, High History	0.0687	0.1212	-0.868	28.9
	Adenoma, No History	0.5261	0.8417	-0.177	9.0
	Adenoma, Low History	0.3570	0.8431	-0.314	14.4
	Adenoma, High History	0.0086	0.0699	-0.880	48.7
Control High History	Adenoma, No History	0.1537	0.0535	0.451	20.1
	Adenoma, Low History	0.3258	0.1405	0.357	14.7
	Adenoma, High History	0.2120	0.6629	-0.410	20.5
Adenoma No History	Adenoma, Low History	0.6942	0.9115	-0.109	5.5
	Adenoma, High History	0.0185	0.0376	-0.807	40.2
Adenoma Low History	Adenoma, High History	0.0442	0.0699	-0.680	34.9
Advanced Adenoma No History	Advanced Adenoma, High History	0.0522	0.0841	-0.698	37.1

Supplemental Figure Legends

SI Figure 1)

Box plots showing normalized L_d values increasing based on the patient's risk history for (a) current controls patients, (b) current adenoma patients, and (c) current advanced adenoma patients.

SI Figure 2)

Additional testing of the effects of confounding factors on L_d. (a) Scatter plot comparing normalized L_d and age for all patients (R^2=0.03). Bar graphs showing averaged normalized L_d

values with patients grouped based on (b) gender, (c) smoking status, and (d) drinking status. The low R^2 and non-significant p-values is further evidence that L_d is not affected by these confounding factors.

SI Figure 3)

This normal probability plot for control patients, no history (n=95) shows that the L_d distribution is slightly skewed to the left. This plot combined with the Anderson-Darling test reported above indicates that the data analyzed in this study is not perfectly normally distributed, but it is close to a normal distribution.

SI Figure 4)

Scatter plot comparing patient L_d values analyzed (cell selection and drawing ROIs) by two different investigators (R^2=0.97). This strong correlation suggests that the results of this study are independent of the investigator analyzing the data.

SI Table 1)

Full set of group comparisons. This table reports the p-value of the Student's t-test, p-value of Mann-Whitney U test, effect size, and percent difference for all group comparisons.

Supplemental Figures

SI Figure 1.

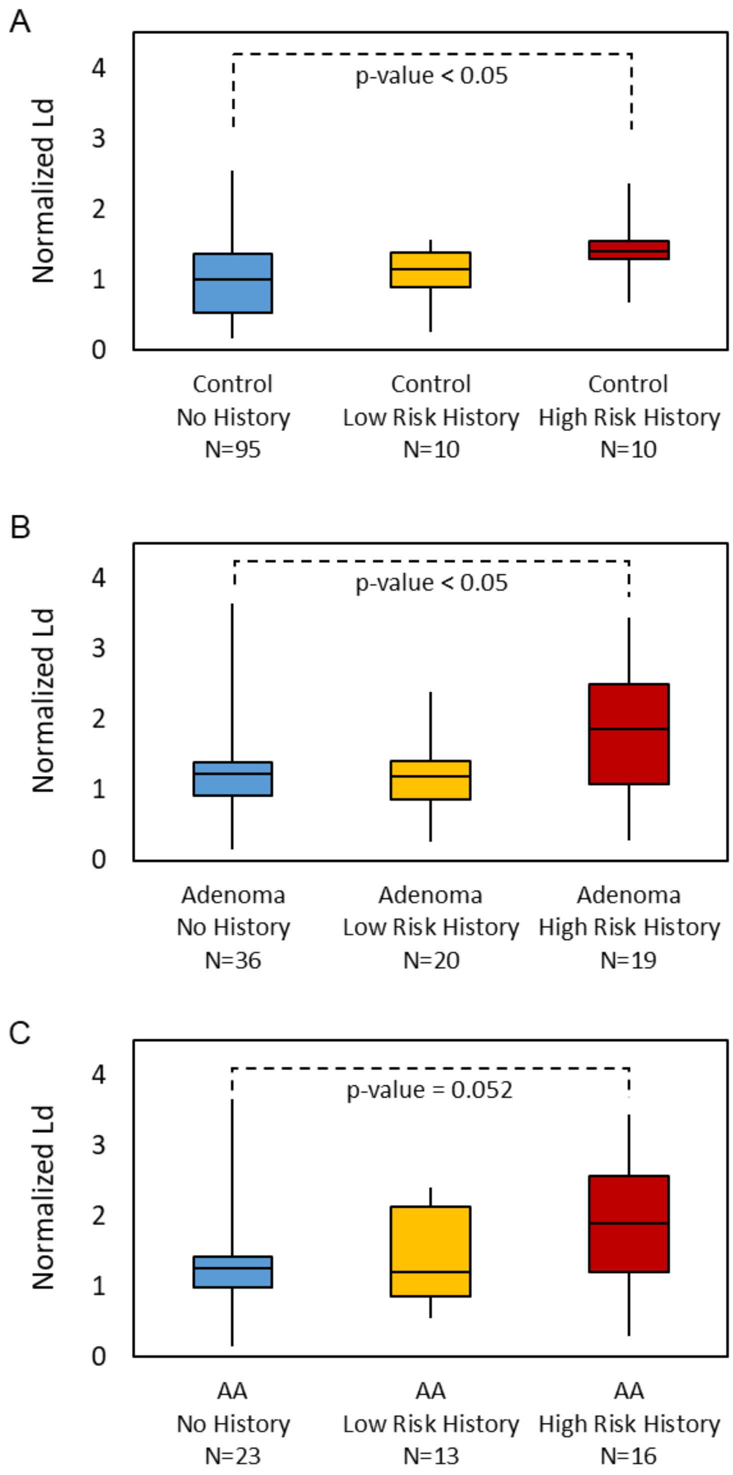

SI Figure 2.

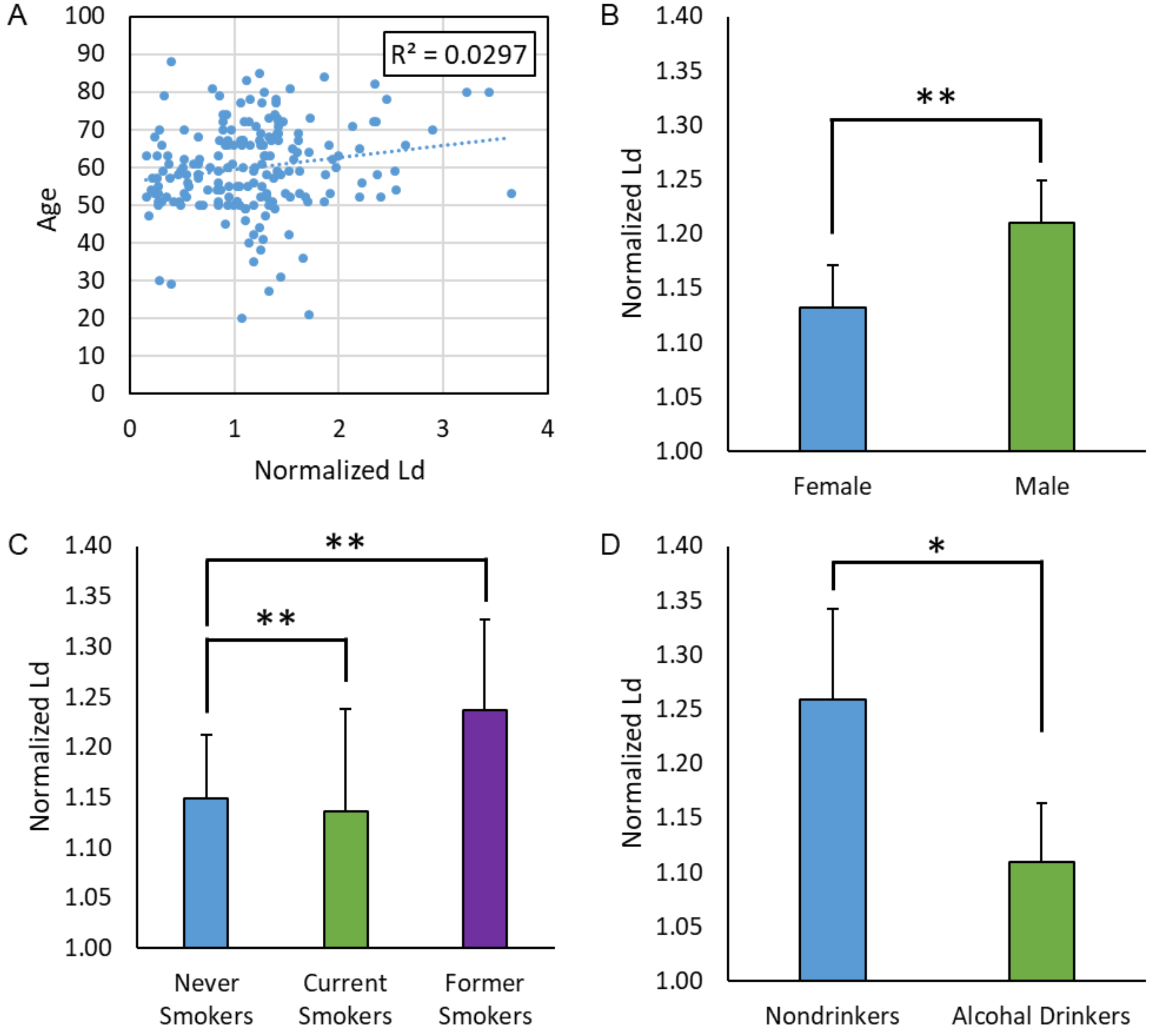

** p-value > 0.4

*   p-value > 0.1

SI Figure 3.

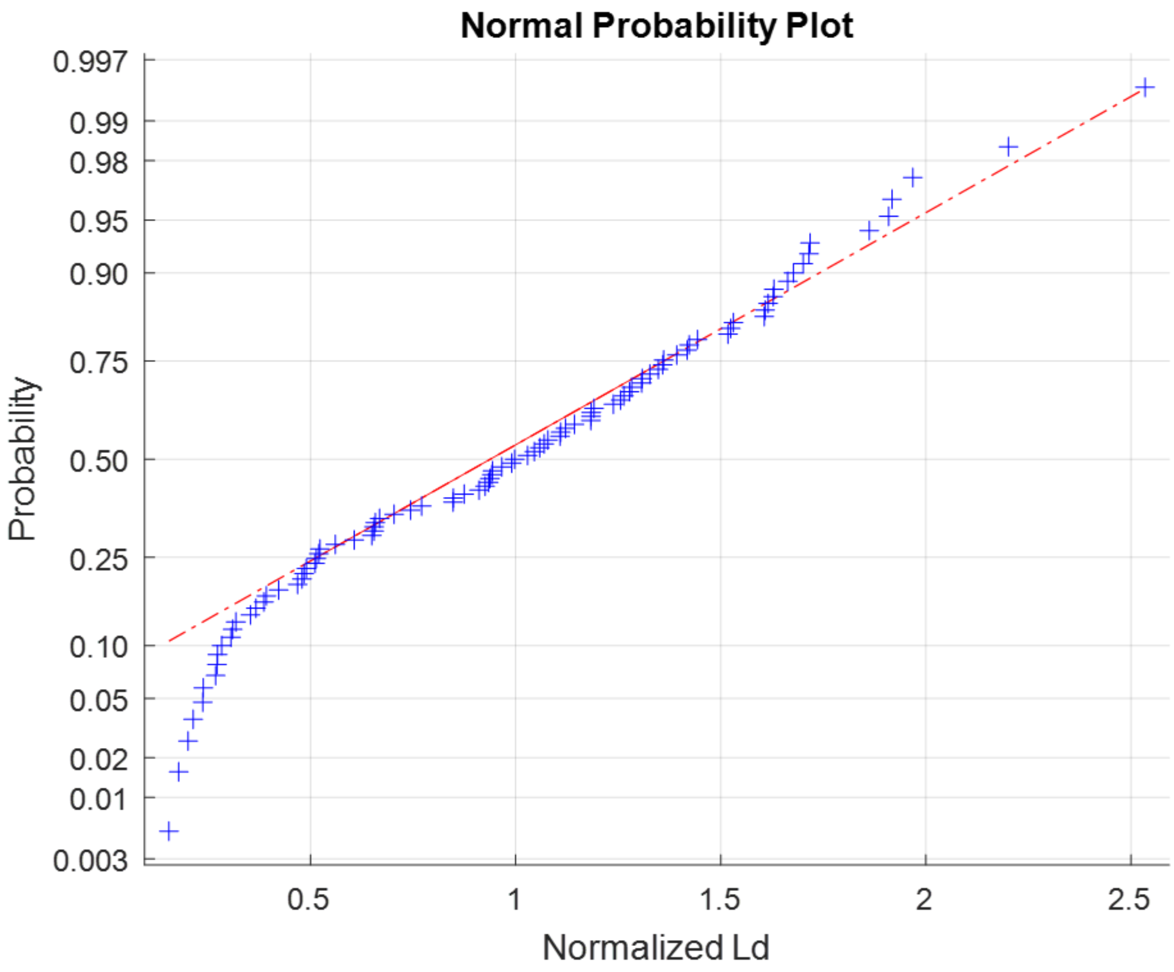
Normal Probability Plot
Probability
0.997
0.99
0.98
0.95
0.90
0.75
0.50
0.25
0.10
0.05
0.02
0.01
0.003
0.5
1
1.5
2
2.5
Normalized Ld

SI Figure 4.

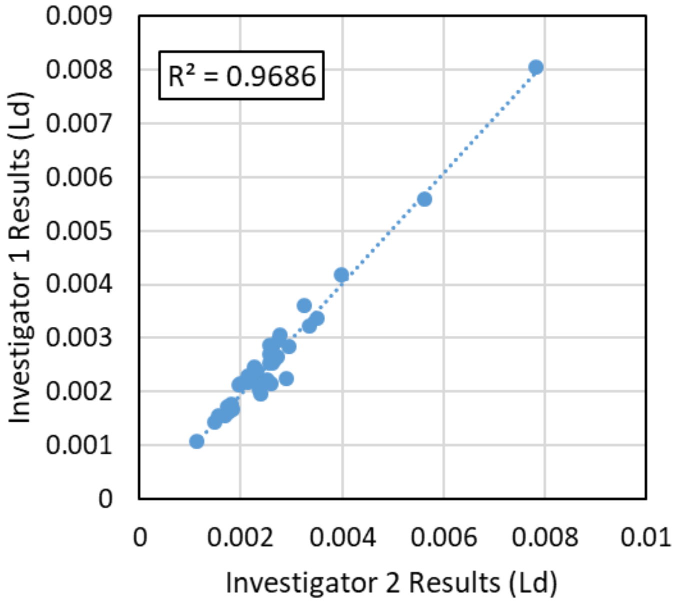
R² = 0.9686
Investigator 1 Results (Ld)
0.009
0.008
0.007
0.006
0.005
0.004
0.003
0.002
0.001
0
0
0.002
0.004
0.006
0.008
0.01
Investigator 2 Results (Ld)
